# Test-retest reliability of health behavior items in the Community Health Survey in South Korea

**DOI:** 10.4178/epih/e2015045

**Published:** 2015-10-20

**Authors:** Soo Jeong Kim, Jin A Han, Young Hwa Kim, Bo Youl Choi, Su Young Kim, Hun Je Lee, In Hwan Oh, Sung-il Cho, Jakyoung Lee, Soon Young Lee

**Affiliations:** 1 Department of Health Administration, Division of Health Sciences, Dongseo Univeresity, Busan, Korea; 2 Center for Radiological, Environmental, and Health Science, Dongseo University, Busan, Korea; 3 Department of Preventive Medicine and Public Health, Ajou University School of Medicine, Suwon, Korea; 4 Department of Preventive Medicine, Hanyang University College of Medicine, Seoul, Korea; 5 Institute for Health and Society, Hanyang University, Seoul, Korea; 6 Department of Preventive Medicine, Jeju National University School of Medicine, Jeju, Korea; 7 Department of Preventive Medicine, Inha University School of Medicine, Incheon, Korea; 8 Department of Preventive Medicine, Kyung Hee University School of Medicine, Seoul, Korea; 9 Graduate School of Public Health and Institute of Health and Environment, Seoul National University, Seoul, Korea; 10 Gyeonggi Center for Hypertension and Diabetes, Suwon, Korea

**Keywords:** Reliability, Health behavior, Kappa, Health surveys

## Abstract

**OBJECTIVES:**

Responses to health-related items on the Community Health Survey (CHS) provide evidence that is used to develop community-based health policy. This study aimed to assess the test-retest reliability of selected health behavioral items on the CHS according to item category, response period, and response scale.

**METHODS:**

A sample of 159 men and women 20 to 69 years of age participated in a test-retest with an interval of 14 to 21 days. A total of 28 items relating to smoking, alcohol consumption, diet and weight control, and mental health were selected. We evaluated the test-retest reliability of the items using kappa statistics.

**RESULTS:**

Kappa values ranged from 0.44 to 0.93. Items concerning habits had higher kappa values (mean, 0.7; standard error, 0.05) than items concerning awareness or attitudes (p=0.012). The kappa value of items with two- to four-point scales was 0.63, which was higher than the value of 0.59 for items with scales involving five or more points, although this difference was not statistically significant. Different kappa values were observed for each reference period, but no statistically significant differences were noted.

**CONCLUSIONS:**

The test-retest reliability of the CHS items that we studied was associated with item category. Further study of the relationship between item category and reliability in domains other than health behaviors is required.

## INTRODUCTION

In many developed countries, efforts have been made to establish health-related policies and to assess the health status of the population through health surveys on the national and community levels. In Korea, the Korea National Health and Nutrition Examination Survey (KNHANES) and the Korea Youth Risk Behavior Web-Based Survey have been carried out on the national level, and the Community Health Survey (CHS) has been carried out on the regional level.

The results from these surveys provide information about major health indices on both the national level and the regional level, and serve as an important information resource for establishing priorities for public health programs, assessing the effectiveness of existing programs, and establishing new health policies. It is thus very important to verify the reliability of the survey questionnaire items, including newly added items, through statistical studies [1-5].

CHS generates regional health statistics used in the implementation of evidence-based health services. In 2008, 2009, and 2010, the national CHS included 360, 300, and 260 core items, respectively. A rotating sampling system was incorporated in the second CHS and is planned to be used in the third CHS (2014 to 2017) [6]. Newly developed indices and items have been added to the CHS, with the goal of further developing the item bank [7].

Previous studies have examined the reliability of selected items in various items and have also tested the reliability of verified items in other populations. In Korea, the reliability of the KNHANES items involving smoking, health-related quality of life, the frequency of food consumption, and food security has been verified [8-10]. Additionally, the items measuring smoking prevalence among students in junior and high schools nationwide have been tested for reliability [11]. Each item related to health behaviors was found to provide independent information, and the scale used for each item varied depending on the characteristics of the item. The existing studies on the reliability of the health behavior questionnaire examined the reliability distribution of the items themselves [11-13].

Although variability in response agreement might occur due to the quality of the interviewer or recall bias, variability according to the category of items can also occur. Therefore, it is important to understand how the interpretation of results might be affected by variability in test-retest reliability according to item category. Few studies have assessed differences in test-retest reliability according to item category, reference period, and response scale.

The purpose of this study was to examine the test-retest reliability of 28 selected CHS items related to health behaviors, with a focus on differences in reliability according to item category, reference period, and response scale.

## MATERIALS AND METHODS

### Subjects and data collection

In order to assess test-retest reliability, four communities were selected out of those included in the CHS through random sampling. Considering our limited budget, the sample size was set at 140 subjects, which was the minimum number of participants that would not affect the evaluation of confidence according to G*Power 3.1.9 version (effect size [F]<0.3; α error=0.05, power=0.8). However, in light of the possibility that some subjects would not undergo retesting, a total of 160 subjects were recruited. The four communities sampled in this study included two urban areas and two rural areas. Forty subjects were drawn from each community, including 20 men and 20 women, with the goal of reflecting the gender and age distribution of the population. In each group, four participants were under 40 years of age, four were in their forties, six were in their fifties, and six were in their sixties.

The study was carried out after explaining its purpose and obtaining consent for further participation in the study from subjects who completed the regular CHS in 2013. The CHS involves two stages of sampling (extracting primary sample points and extracting sample households). The present study chose four regions (two urban and two rural areas), with consideration of the quality of the investigators and the level of collaboration from the investigated sites, and used the extant sampling frame of the CHS to select household subjects. The study subjects included one to seven people per household. The final subjects were categorized by gender and age, and were sampled by convenience. The interviews were conducted in no special order, and all adults 20 years of age or older in a household were included as subjects. The 160 subjects who agreed to participate in the study were part of the sample recruited for the CHS in 2013 from four communities. Two trained survey interviewers conducted one-on-one interviews with 20 subjects in each of the four communities. Each interviewer explained the purpose of the study and asked subjects to sign a consent form for the study. The survey period extended from September 1 to September 14, 2013. The retest period was September 15 to October 5, 2013. The retest was scheduled to be administered 14 to 21 days after the first interview. The follow-up interviews were conducted by the same investigator that conducted the initial interview. The study protocol was approved by the institutional review board of Seoul National University.

### Criteria for selecting items

Sixteen of the 28 items included in the present study were core items (i.e., nationwide common items), and 12 items were chosen from optional survey items [7]. Items were excluded based on the following criteria: 1) items with a predicted response rate close to 0%, 2) items for which respondents might change their behavior in two weeks, and 3) items with learning effects. The final 28 items selected involved smoking (five items), alcohol consumption (four items), safety (two items), physical activity (five items), diet and weight control (four items), and mental health (eight items). Five of the items measuring mental health were drawn from the Brief Encounter Psychosocial Instrument, which assesses stress levels.

### Item characteristics

The selected items were categorized depending on whether they assessed habits, awareness, or attitudes. For example, an item asking “Do you currently smoke?” was classified as a habit item, an item asking “Do you know about designated smoking areas?” as an awareness item, and an item asking “Are you planning to quit smoking?” as an attitudes item. The reference periods were “now,” “usually,” “one week,” “one month,” “one year,” “lifetime,” and “future.” The response scales ranged from two to eight points. For our analysis, items were reclassified into two-point to four-point scales and scales with five or more points.

### Statistical analysis

Reliability implies that an instrument or a questionnaire produces consistent results from the same respondents [14]. In order to test the reliability of the CHS, the simple kappa coefficient, which was introduced by Landis & Koch [15], was employed to measure the agreement between two raters for 2×2 tables. The relative importance of disagreement between categories may not be the same for adjacent categories as it is for distant categories. For tables larger than 2×2, the weighted kappa coefficient suggested by Fleiss & Cohen [16] was used. A kappa value of 0.81 or more indicates almost perfect agreement, while values from 0.61 to 0.80 indicate substantial agreement. Values from 0.41 to 0.60 indicate moderate agreement, and values from 0.21 to 0.40 indicate fair agreement. Values from 0 to 0.20 indicate slight agreement, while values less than 0 indicate no agreement [15].

A general frequency analysis of the demographic characteristics of the participating subjects was conducted. Reliability depending on the characteristics of the selected items, reference period, and response scale were examined using simple kappa and weighted kappa. Differences in kappa or weighted kappa according to the characteristics of the items, reference period, and response scale were analyzed using one-way ANOVA and the independent t-test. All statistical analyses were performed using SAS version 9.4 (SAS Institute Inc., Cary, NC, USA). All p-values were two-tailed, and p-values<0.05 were considered to indicate statistical significance.

## RESULTS

A total of 160 subjects participated in the first interview, and 159 subjects participated in the follow-up interview. Thus, 159 subjects were included in the statistical analysis. Table 1 shows the general demographic characteristics of the subjects. The gender ratio was approximately equal, with 50.3% of the sample composed of men. Subjects ranged in age from 20 to 69 years old. The mean age was 50.6±12.6 years old, 59.8% of the respondents were above 50 years of age, 89.3% of the subjects were married (120 subjects lived with their spouse, two lived without their spouse, nine had experienced the death of their spouse, and 11 subjects were divorced), and 38.4% reported 13 or more years of education.

Three of the 28 items showed a kappa value greater than 0.81,indicating almost perfect agreement, 10 items showed kappa values ranging from 0.61 to 0.80, indicating substantial agreement, while 15 items showed moderate agreement (Appendix 1). Table 2 shows differences in reliability according to item category, reference period, and response scale. Items concerning habits had higher kappa values than items concerning awareness or attitudes (p=0.012), but no significant differences in kappa values were found according to the reference period. The kappa value of two-point to four-point scales was 0.63, which was higher than the kappa value of 0.59 observed for items with a scale of more than five points, but this difference was not statistically significant.

Additionally, we analyzed the mean differences between the kappa values of all 28 items depending on the respondents’ characteristics. No significant differences in the mean kappa values were found according to gender (men vs. women), age (below 50 years old vs. above 50 years old), and years of education (12 years and below vs. 13 years and above) (Table 3).

## DISCUSSION

The study aimed to examine the test-retest reliability of the health behavior items used in the CHS, which was conducted via individual interviews with adults in selected communities. Our study examined differences in reliability according to item category, reference period, and response scale.

The highest kappa and weighted kappa scores were observed in items assessing habits, followed by those assessing attitudes and awareness. Since habits are repetitive behaviors, they may be more clearly remembered than levels of awareness and attitude. Attitudes can change within two weeks, and cognition depends on memory, which can also change within two weeks. A study of 11 to 15-year-old students in China used intraclass correlation coefficients (ICCs) to examine the reliability of 33 items involving physical activity, sedentary behavior, sleeping, and risky behaviors, such as smoking and drinking, within a three-week test-retest interval. Items involving smoking and drinking behaviors showed little change over time; however, other items asking about everyday life behaviors showed significant changes. The authors suggested that recalling behaviors such as smoking and drinking was easier than recalling behaviors involving physical activity, sedentary behavior, and sleeping. The ICC values were very low for sedentary behavior (i.e., computer use or TV watching). The authors speculated that sedentary behaviors might be dependent on school programs and situations [12]. In a study of the test-retest reliability of items related to health behaviors among students in middle and high school in Korea, the reliability value was different for each category. Items involving important health risk factors, such as smoking, showed higher reliability, while items assessing situation-dependent everyday behaviors, such as hand washing, had low reliability [13].

The lifetime reference period had the highest kappa value, which is consistent with the results of other studies [13,17]. Recalling a certain behavior over a limited time requires a more complex cognitive process than recalling a behavior over one’s lifetime [13]. In our literature search, studies examining test-retest reliability according to response scale were limited. In this study, reliability was slightly higher in the items with scales involving five or more points than in items with two-point to four-point scales, but this difference was not statistically significant.

No significant differences in the mean kappa value were found according to gender, age group, or education level. However, this result could not be compared with those of other studies, because it was difficult to find papers analyzing differences in test-retest reliability according to demographic characteristics. The current study examined only 28 items. Therefore, although a univariate analysis showed the presence of certain correlations, it was not possible to examine the magnitude of the influence of each independent variable through regression analysis. Nevertheless, this is a significant study of differences in test-retest reliability according to the characteristics of survey items related to health behaviors. Future studies should be conducted to test the reliability of other items according to item characteristics, reference period, and response scale.

## Figures and Tables

**Table 1.** Demographic characteristics of the respondents

Characteristics		n	%
Region	Guri	40	25.2
	Gwachon	39	24.5
	Namyangju	39	24.5
	Yonchon	41	25.8
Gender	Men	80	50.3
	Women	79	49.7
Age (yr)	<40	31	19.5
	40-49	33	20.8
	50-59	48	30.2
	≥60	47	29.6
Marital status	Married^1^	142	89.3
	Unmarried	17	10.7
Education (yr)	≤12	98	61.6
	≥13	61	38.4

1 One hundred and twenty subjects lived with their spouse, two lived without their spouse, nine had experienced the death of their spouse, and 11 subjects were divorced.

**Table 2.** Reliability of items by category, reference period, and response scale

		No. of items	Kappa				p-value^1^
			Mean	Standard error	Minimum	Maximum	
Item category	Habits	9	0.70	0.05	0.51	0.93	0.012
	Awareness	13	0.52	0.01	0.44	0.67	
	Attitudes	6	0.55	0.04	0.45	0.72	
Reference period	Now	8	0.64	0.06	0.47	0.93	0.57
	Usually	3	0.52	0.04	0.45	0.58	
	One week	3	0.58	0.07	0.53	0.66	
	One month	5	0.55	0.07	0.44	0.61	
	One year	5	0.62	0.08	0.50	0.68	
	Lifetime	2	0.70	0.19	0.51	0.88	
	Future	2	0.69	0.04	0.66	0.72	
Response scale	2-4	15	0.63	0.04	0.47	0.93	0.43
(point)	≥5	13	0.59	0.03	0.44	0.82	

1 Independent t-test or one-way analysis of variance.

**Table 3.** Kappa statistics by characteristics of the respondents

		Mean^1^	Standard error	Minimum	Maximum	p-value
Region	Guri	0.62	0.03	0.38	1.00	0.15
	Gwachon	0.54	0.03	0.19	0.97	
	Namyangju	0.57	0.03	0.16	0.94	
	Yonchon	0.64	0.02	0.38	0.90	
Gender	Men	0.61	0.02	0.38	0.96	0.25
	Women	0.57	0.02	0.41	0.92	
Age (yr)	<50	0.60	0.02	0.37	0.84	0.84
	≥50	0.51	0.03	0.32	1.00	
Education (yr)	≤12	0.61	0.03	0.37	0.93	0.60
	≥13	0.59	0.03	0.26	0.93	

1 Mean kappa value of all 28 items.

## Appendix 1. Test-retest reliability of selected items of Community Health Survey questionnaire

**Table:**

Domains	Characteristics	Reference periods	Items	Response scales	Response alternatives	Kappa^†^ or weighted kappa
Smoking	Habit	Lifetime	Have you smoked more than 5 packs of cigarette in your life?	2	Yes, no	0.88^†^
	Habit	Now	Are you currently smoking?	3	Every day, sometimes, quit smoking	0.93
	Attitude	Future	Do you have a plan to quit smoking?	4	Plan to quit within a month; Plan to quit smoking within 6 months; Plan to quit someday but not within 6 months	0.71
	Awareness	One year	Have you ever seen or heard of anti-smoking campaign by health department during the past 1 year?	2	Yes, no	0.50^†^
	Awareness	Now	Are you aware of the designated smoke free areas?	3	Aware of the designated smoke free areas and know where they are; aware of the designated smoke free areas but do not know where they are; not aware of the designated smoke free areas	0.53
Drinking	Habit	Lifetime	Have you ever drunk more than one glass of alcohol beverages?	2	Yes, no	0.51^†^
	Habit	One year	Have you ever drunk an alcohol beverage during the past one year?	2	Yes, no	0.68^†^
	Habit	Now	How often do you drink alcohol beverage?	5	Less than once a month; once a month; two to three times a month; two to three times a week; more than four times a week	0.74
	Habit	Now	How much do you drink alcohol beverage when you drink?	5	1-2 glasses; 3-4 glasses; 5-6 glasses; 7-9 glasses; more than 10 glasses	0.82
Safety	Awareness	Now	Do you know #1339 or #119 as 24 hour call numbers to get a professional help from health professionals in case of emergency?	2	Yes, no	0.50^†^
	Awareness	Now	Do you know about School -Zone (Areas designated for Protecting children)?	2	Yes, no	0.47^†^
Exercise	Habit	One week	How many hours have you spent for TV watching, game, and internet use for your leisure time during the weekdays in the last week?	5	Less than one hour per day; less than 1-2 hours per day; less than 2-3 hours per day; less than 3-4 hours per day; more than 4 hours per day	0.56
	Habit	One week	How many hours did you spent for TV watching, game and internet for your leisure time during the weekend in the last week?	5	Less than one hour per day; less than 1-2 hours per day; less than 2-3 hours per day; less than 3-4 hours per day; more than 4 hours per day	0.53
	Attitude	Usual	In your opinion, do you think it is necessary to exercise for your health?	5	Strongly agree, agree, neutral, disagree, strongly disagree	0.45
	Attitude	Future	Do you have a plan to exercise on a regular basis?	4	Plan to exercise within a month; plan to exercise within 6 months; plan to exercise someday but not within 6 months; no plan to exercise	0.66
	Awareness	One year	Have you seen or heard of exercise campaign during the past 12 months?	2	Yes, no	0.65^†^
Nutrition	Habit	One week	How many days did you have a breakfast during the last 7 days?	8	0-7 days	0.66
	Attitude	Now	Do you think it is necessary to have a low sodium diet education?	5	Strongly agree, agree, neutral, disagree, strongly disagree	0.51
	Awareness	Now	How do you feel about your body shape?	5	Very skinny, a little skinny, average, a little overweight, very much overweight	0.65
	Attitude	One year	Have you ever tried to lose weight during the past 12 months?	4	Tried to lose weight; tried to maintain the weight; tried to gain weight; not tried to lose or gain weight	0.58
Mental health	Awareness	Usual	How much stressful are you?	4	Very much stressed; stressed, a little bit stressed, hardly stressed	0.52
	Attitude	Usual	Do you try to manage your stress?	2	Yes, no	0.58^†^
	Awareness	One month	In the fast month have you ever felt as if there are more demands in your life, emotionally and physically, than you can handle comfortably?	5	Never, rarely, frequently, almost always, always	0.44
	Awareness	One month	In the fast month, have you ever felt frustrated trying to live up to your own expectations or standards?	5	Never, rarely, frequently, almost always, always	0.61
	Awareness	One month	In the fast month, have you ever felt that your needs as a person are being left environment?	5	Never, rarely, frequently, almost always, always	0.61
	Awareness	One month	In the fast month, have you ever felt uncertain or apprehensive about the future	5	Never, rarely, frequently, almost always, always	0.55
	Awareness	One month	In the fast month, have you ever felt that there are so many everyday hassles and crises that you lose track of the things that are really important to you?	5	Never, rarely, frequently, almost always, always	0.55
	Awareness	One year	Have you ever think about committing a suicide?	2	Yes, no	0.67^†^
